# Fifteen-year temporal changes in rates of acute kidney injury among children in Denmark

**DOI:** 10.1007/s00467-023-06246-9

**Published:** 2023-12-18

**Authors:** Sidse Høyer, Uffe Heide-Jørgensen, Simon Kok Jensen, Mette Nørgaard, Cara Slagle, Stuart Goldstein, Christian Fynbo Christiansen

**Affiliations:** 1 Department of Clinical Epidemiology and Department of Clinical Medicine, Aarhus University Hospital and Aarhus University, Aarhus, Denmark; 2 Department of Pediatrics, University of Cincinnati, Cincinnati, OH USA; 3 Center for Acute Care Nephrology and Division of Neonatal and Pulmonary Biology, Cincinnati Children’s Hospital Medical Center, Cincinnati, OH USA; 4 Center for Acute Care Nephrology, Cincinnati Children’s Hospital Medical Center, Cincinnati, OH USA

**Keywords:** Acute kidney injury, Pediatric nephrology, Epidemiology, Temporal trends

## Abstract

**Background:**

We aimed to examine temporal changes in the annual rate of acute kidney injury (AKI) in Danish children and associated changes in patient characteristics including potential underlying risk factors.

**Methods:**

In this population-based cohort study, we used plasma creatinine measurements from Danish laboratory databases to identify AKI episodes in children aged 0–17 years from 2007 to 2021. For each child, the first AKI episode per calendar year was included. We estimated the annual crude and sex- and age-standardized AKI rate as the number of children with an AKI episode divided by the total number of children as reported by census numbers. Using Danish medical databases, we assessed patient characteristics including potential risk factors for AKI, such as use of nephrotoxic medication, surgery, sepsis, and perinatal factors.

**Results:**

In total, 14,200 children contributed with 16,345 AKI episodes over 15 years. The mean annual AKI rate was 148 (95% CI: 141–155) per 100,000 children. From 2007 to 2021, the annual AKI rate demonstrated minor year-to-year variability without any discernible overall trend. The highest AKI rate was recorded in 2007 at 174 (95% CI: 161–187) per 100,000 children, while the lowest rate occurred in 2012 at 129 (95% CI: 118–140) per 100,000 children. In 2021, the AKI rate was 148 (95% CI: 141–155) per 100,000 children. Characteristics of children with AKI were similar throughout the study period.

**Conclusion:**

The rate of AKI among Danish children was stable from 2007 to 2021 with little variation in patient characteristics over time.

**Graphical abstract:**

**Figure:**
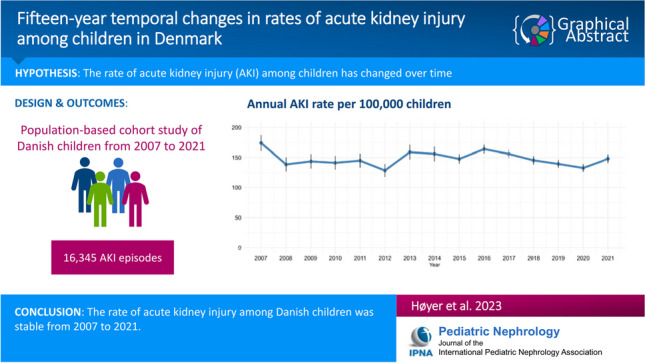
A higher resolution version of the Graphical abstract is available as Supplementary information

**Supplementary Information:**

The online version contains supplementary material available at 10.1007/s00467-023-06246-9.

## Introduction

Acute kidney injury (AKI) is defined as a sudden decrease in kidney function and is associated with substantial morbidity and mortality [1, 2]. In children, AKI is most frequent among infants and adolescents, and approximately 20% of all pediatric AKI episodes occur among children ≤ 28 days of age (neonates) [3]. Recent studies suggest a prevalence of pediatric AKI of 27% in the intensive care unit (ICU) [2] and at least 5% among hospitalized children in a non-ICU setting [4]. Nevertheless, reported AKI rates demonstrate significant variation, largely due to differences in population characteristics and the methods used to define AKI [2, 4, 5]. Only few studies have reported AKI rates at a population-level, with one such study revealing an incidence of 137 per 100,000 person-years among children below 18 years of age [5]. However, most prior studies have primarily focused on critically ill children experiencing their first AKI episode [2, 6], and large population-based studies on AKI rates are lacking. Most, but not all, studies have suggested that the AKI incidence among the adult population is increasing over time [7–10]; however, it is uncertain if this trend reflects an actual increase in AKI incidence or is due to an increased AKI awareness. Furthermore, it remains unclear whether a similar trend pertains to children. Any change in AKI incidence may originate from a time-varying prevalence of risk factors for AKI [11–14]. In children, AKI risk factors include use of nephrotoxic medication, surgery, and sepsis. For neonates in particular, low birth weight, preterm birth, and birth defects increase the risk of AKI [1, 15, 16]. Any potential increasing trend in AKI incidence and related risk factors is critical knowledge for AKI prevention, allocation of healthcare resources, and future research. In this population-based study, we aimed to examine temporal changes in the annual rate of AKI in Danish children, and to describe associated changes in patient characteristics.

## Methods

### Setting and design

In this population-based cohort study, we included Danish children aged 0–17 years in the period 2007 to 2021. We included AKI episodes from the Danish laboratory databases since 2007 because the method used to estimate plasma creatinine (pCr) has been largely consistent since then [17]. A unique identification number is assigned to all citizens in Denmark at birth or immigration, which allows individual-level linkage between the Danish medical databases. The treatment of children with acute illness usually occurs in the public healthcare sector, and the expenses associated with their care are covered by the tax-funded healthcare system [18]. Healthcare data including biochemical test results, diagnoses, and performed procedures are reported to nationwide registries. In this study, data on children with AKI were linked to information from the Danish Civil Registration System, the Danish National Patient Registry, the Danish National Prescription Registry, and the Danish Medical Birth Register [19–24].

### Study population

We identified children with AKI (as described below) from the Danish National Patient Registry and from two Danish laboratory databases: The Laboratory Information System (LABKA, covering the Central Denmark Region and the North Denmark Region) and the Register of Laboratory Results for Research (RLRR, covering all Danish regions). LABKA and the RLRR contain information on blood samples taken in hospitals or general practice [19, 20]. The laboratory databases achieved complete reporting from the Danish municipalities at varying times, which has been described in detail elsewhere [25]. In this study, we restricted data to areas with complete laboratory coverage when defining the study population of children at risk. As a result, our study population expanded from 378,931 children in 2007 to 1,152,995 children in 2021. We obtained the count of children each study year from Statistics Denmark, which provides annually updated information on the number of children with Danish residence [26]. To ensure consistency, we included only the first AKI episode per calendar year for each child (the first annual AKI episode). Exclusion criteria were non-Danish residency, dialysis-requiring chronic kidney disease, and previous kidney transplantation.

### Acute kidney injury

We applied the Kidney Disease: Improving Global Outcomes (KDIGO) pediatric/neonatal guidelines on all available creatinine tests to define AKI (Supplementary Table S1). These guidelines require an absolute increase of ≥ 26.5 µmol/L in pCr within the previous 48 h or a ≥ 1.5 times increase in pCr from baseline [1, 27]. We did not require that the index pCr reached a specific threshold. For neonates, the baseline was defined as the lowest previous pCr. For other children, baseline pCr was defined as the lowest inpatient or outpatient pCr within the previous 7 days, provided that a pCr test was performed in this period; otherwise, it was defined as the median outpatient pCr at 8–90 days before the AKI. If a baseline pCr was not available, the Pottel-age-based imputation method was used to estimate baseline pCr [28]. This approach was taken to prevent underestimation of the AKI rate. For children < 2 years of age or those with a pre-diagnosed chronic kidney disease, we only included lab-identified AKI episodes without a baseline pCr if they were confirmed by a relevant ICD-10 code. We staged AKI severity by the highest obtained pCr within 30 days after the AKI episode and according to the pediatric/neonatal KDIGO guidelines (Supplementary Table S1). To ensure inclusion of incident AKI episodes, we defined an AKI episode as lasting 30 days during which no new AKI episodes was considered, a criterion also applicable to AKI episodes occurring over the turn of the calendar year. We categorized AKI episodes as hospital-acquired episodes when the defining pCr was taken ≥ 1 day after admission, and community-acquired episodes when pCr was taken in the outpatient clinic or at the first day of admission [29]. No information on urine output was available.

### Covariates

At the time of fulfilling the AKI definition, we obtained baseline characteristics of the child, including age, sex, comorbidities, recent risk factors (diagnosed kidney disease, infection, sepsis, surgery, and use of potential nephrotoxic medication dispensed from community pharmacies), and perinatal factors (birth weight and gestational age). Data on these covariates were obtained from the Danish Civil Registration System (which holds information on e.g., sex, date of birth, vital status and residence of all individuals in Denmark), the National Patient Registry (which holds information on e.g., admissions, diagnoses, medical treatments, and surgical procedures), the Danish National Prescription Registry (which holds information on all dispensed prescriptions at community pharmacies), and the Medical Birth Register (which holds information on maternal health, delivery, and infant outcomes). All comorbidities were defined by ICD-10 coded hospital diagnoses registered within five years before the AKI, except birth defects which could be registered any time before the AKI (Supplementary Table S2). Recent risk factors were identified up to three months before the AKI episodes (Supplementary Table S3). We defined low birth weight as birth weight < 2500 g and preterm birth as being born < 37 gestational weeks. Based on data from the Medical Birth Register, we grouped weight for gestational age into ≤ 10^th^ percentile (small for gestational age, SGA), 10^th^–90^th^ percentile (appropriate for gestational age, AGA), and ≥ 90^th^ percentile (large for gestational age, LGA).

### Statistical analyses

Categorical data are presented as counts with percentages and continuous data as medians with interquartile ranges. The annual rate was computed as the number of children with at least one AKI episode per 100,000 children living in the study area covered by the laboratory database on 1 January of that year, which was assumed to correspond to 100,000 person-years. For direct standardized rates, we applied age- and sex-specific weights derived from the general population in 2015. All rates were provided with 95% confidence intervals (CI). Calendar years were categorized in 3-year intervals when describing baseline characteristics. Analyses were conducted using R statistical software version 4.2.2 [30, 31].

### Additional analyses

We examined temporal changes in pCr test frequency by presenting the number of children with at least one creatinine test per year as absolute counts, and count per 100,000 children covered by the laboratory databases. We performed four sensitivity analyses for the annual AKI rate: 1) stratified according to age and sex; 2) restricted to AKI episodes based on a known baseline pCr; 3) restricted to the study area covered by the laboratory databases throughout the study period; and 4) including additional AKI episodes defined by point-of-care-testing (POCT) of creatinine. Measurements by this method were omitted from the primary analysis because they were not consistently utilized over the 15-year time period.

### Ethical approval

The study was registered at Aarhus University (record number 2016–051-000001/812). Data access and approval were obtained by the Danish Health Data Authority (FSEID-00003631). According to Danish legislation, ethical approval was not required.

### Data availability

Due to Danish legislation, the dataset used in the present study is not publicly available, and the sharing of patient data with external parties is restricted. Access to the data is granted by the Danish Health Authority.

## Results

### AKI rates

**Fig. 1:**
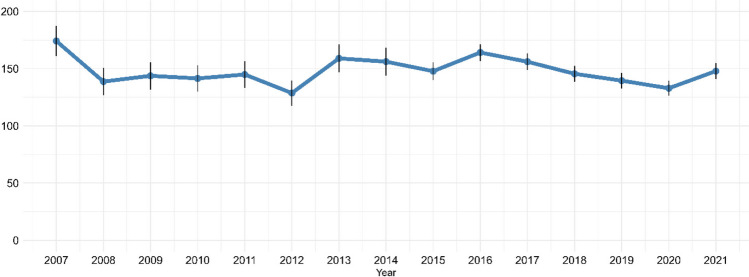
Annual AKI rate per 100,000 children

**Fig. 2:**
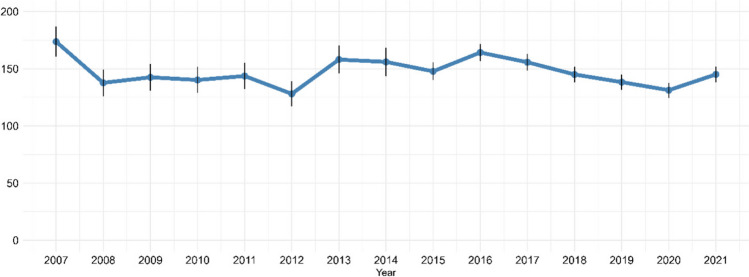
Age- and sex-standardized AKI rate per 100,000 children

In total, 14,200 children contributed with 16,345 AKI episodes (first episodes per calendar year). Over the period from 2007 to 2021, the annual AKI rate remained relatively stable with only minor fluctuations, and no clear overall trend was observed (Fig. 1). The average annual AKI rate throughout the study period was 148 (95% CI: 141–155) per 100,000 children. The AKI rate ranged from 129 (95% CI: 118–140) per 100,000 children in 2012 to 174 (95% CI: 161–187) per 100,000 children in 2007, and the most recent rate in 2021 was 148 (95% CI: 141–155) per 100,000 children. We observed little change in demographics during the study period, therefore the corresponding annual age- and sex-standardized AKI rate exhibited virtually identical fluctuations, with an average of 147 (95% CI: 140–154) per 100,000 children (Fig. 2).

### Characteristics of the AKI episodes

Overall, AKI episodes were more frequently stage 1 (77%), community-acquired (66%), and occurring among boys (54%) (Table 1). In 53% of the AKI episodes the baseline pCr was imputed. Most episodes with a baseline pCr were identified based on a pCr within the prior 7 days (34%). We found a high prevalence of cancer in the AKI episodes (11%) and 27% of the AKI episodes occurred in children with a congenital birth defect, in particular congenital heart disease and congenital anomalies of the kidney and urinary tract. Recent risk factors included use of potential nephrotoxic medication in 32% of episodes, a diagnosis of kidney disease in 6% of episodes, sepsis in 2% of episodes, and surgery in 21% of episodes. Of the perinatal factors, low birth weight and preterm birth were found in 9% and 11% of the AKI episodes, respectively. We observed some missing data (~ 7%) for the perinatal factors due to birth in foreign countries or incomplete reporting to the Danish Medical Birth Register. The proportion of missing data increased slightly in the later study years. Patient characteristics were similar throughout the study period, except for minor temporal changes in surgery (increased) and comorbidities such as pulmonary disease (decreased), kidney disease (increased), and birth defects (increased).

**Table 1:** Baseline characteristics of 16,345 AKI episodes from 2007–2021

	Year					
	Total	2007–2009	2010–2012	2013–2015	2016–2018	2019–2021
Number of AKI episodes	16,345	1,745	1,695	2,610	5,435	4,860
Number of children	14,200	1,639	1,452	2,291	4,730	4,088
Number of years at risk per MM	1689.8	62.7	73.5	187.2	680.0	686.2
Demographics						
Age, median years (IQR)	9 (3–15)	8 (3–14)	9 (3–15)	10 (4–15)	10 (4–15)	9 (3–15)
Age category, N (%)						
≤ *28 days*	548 (3)	72 (4)	58 (3)	61 (2)	174 (3)	183 (4)
*29 days –* < *2 years*	2,233 (14)	242 (14)	216 (13)	329 (13)	670 (12)	776 (16)
*2–5 years*	2,810 (17)	349 (20)	311 (18)	463 (18)	864 (16)	832 (17)
*6–9 years*	2,349 (14)	269 (15)	272 (16)	388 (15)	818 (15)	602 (12)
*10–13 years*	2,140 (13)	237 (14)	207 (12)	350 (13)	739 (14)	607 (12)
≥ *14 years*	6,265 (38)	576 (33)	631 (37)	1,019 (39)	2,170 (40)	1,869 (38)
Male, N (%)	8,827 (54)	936 (54)	917 (54)	1,413 (54)	2,967 (55)	2,594 (53)
AKI-defining factors						
Severity of AKI, N (%)						
*Stage 1*	12,605 (77)	1,272 (73)	1,299 (77)	2,085 (80)	4,254 (78)	3,695 (76)
*Stage 2*	2,129 (13)	262 (15)	216 (13)	313 (12)	666 (12)	672 (14)
*Stage 3*	1,611 (10)	211 (12)	180 (11)	212 (8)	515 (9)	493 (10)
AKI based on imputed baseline pCr, N (%)	8,683 (53)	870 (50)	862 (51)	1,427 (55)	3,118 (57)	2,406 (50)
AKI based on a known baseline pCr, N (%)	7,662 (47)	875 (50)	833 (49)	1,183 (45)	2,317 (43)	2,454 (50)
pCr within 48 h	1,053 (6)	135 (8)	111 (7)	158 (6)	321(6)	328 (7)
pCr within 7 days	5,505 (34)	631 (36)	605 (36)	846 (32)	1,659 (31)	1,764 (36)
pCr 8–90 days prior	2,415 (15)	256 (15)	235 (14)	386 (15)	758 (14)	780 (16)
Median pCr (IQR)	30 (20–45)	26 (19–38)	34 (24–49)	30 (21–44)	31 (21–47)	28 (19–44)
Number of tests, median (IQR)	2 (1–4)	1 (1–4)	2 (1–4)	1 (1–3)	2 (1–4)	2 (1–5)
Median days to latest test (IQR)	27 (14–49)	30 (15–51)	27 (14–52)	28 (14–47)	27 (14–50)	26 (14–46)
Location for AKI*, N (%)						
*Community-acquired*	10,799 (66)	1,109 (64)	1,075 (63)	1,748 (67)	3,790 (70)	3,077 (63)
*Hospital-acquired*	5,546 (34)	636 (36)	620 (37)	862 (33)	1,645 (30)	1,783 (37)
Comorbidities, N (%)						
Hypertension	344 (2)	19 (1)	32 (2)	67 (3)	135 (2)	91 (2)
Diabetes	299 (2)	38 (2)	42 (2)	48 (2)	101 (2)	70 (1)
Cancer	1,774 (11)	168 (10)	197 (12)	301 (12)	553 (10)	555 (11)
Kidney disease	515 (3)	28 (2)	48 (3)	66 (3)	201 (4)	172 (4)
Cardiovascular disease	578 (4)	46 (3)	63 (4)	85 (3)	203 (4)	181 (4)
Gastrointestinal disease	689 (4)	62 (4)	62 (4)	99 (4)	260 (5)	206 (4)
Pulmonary disease	1,020 (6)	138 (8)	119 (7)	160 (6)	340 (6)	263 (5)
Birth defects	4,473 (27)	418 (24)	447 (26)	662 (25)	1,527 (28)	1,419 (29)
CAKUT	742 (5)	66 (4)	77 (5)	126 (5)	254 (5)	219 (5)
Congenital heart defect	1,716 (10)	160 (9)	191 (11)	255 (10)	564 (10)	546 (11)
Other birth defect	3,044 (19)	301 (17)	309 (18)	458 (18)	1,048 (19)	928 (19)
Recent risk factors within 3 months, N (%)						
Potential nephrotoxic medication	5,294 (32)	557 (32)	587 (35)	879 (34)	1,781 (33)	1,490 (31)
Analgesics	1,674 (10)	164 (9)	158 (9)	265 (10)	555 (10)	532 (11)
Antimicrobials	3,483 (21)	368 (21)	401 (24)	615 (24)	1,162 (21)	938 (19)
Benzodiazepines	397 (2)	50 (3)	45 (3)	59 (2)	142 (3)	101 (2)
Antihypertensive	685 (4)	73 (4)	90 (5)	109 (4)	218 (4)	195 (4)
Proton pump inhibitor	1,053 (6)	85 (5)	116 (7)	143 (5)	403 (7)	306 (6)
Other	927 (6)	135 (8)	99 (6)	143 (5)	299 (6)	251 (5)
Diagnosed kidney disease	959 (6)	81 (5)	96 (6)	133 (5)	307 (6)	342 (7)
Diagnosed infection	3,558 (22)	425 (24)	404 (24)	600 (23)	1,087 (20)	1,042 (21)
Sepsis	317 (2)	43 (2)	38 (2)	43 (2)	94 (2)	99 (2)
Surgery	3,396 (21)	271 (16)	347 (20)	462 (18)	1,019 (19)	1,297 (27)
Perinatal factors						
Birth weight, median grams (IQR)	3450 (3020–3820)	3450 (3034–3820)	3475 (3051–3890)	3490 (3050–3844)	3420 (3000–3800)	3449 (3020–3818)
Low birth weight, N (%)	1,454 (9)	171 (10)	130 (8)	214 (8)	481 (9)	458 (9)
Missing, N (%)	1,242 (8)	96 (6)	118 (7)	160 (6)	458 (8)	408 (8)
Gestational age, median weeks (IQR)	39 (38–40)	40 (38–40)	39 (38–40)	39 (38–40)	39 (38–40)	39 (38–40)
Preterm birth, N (%)	1,751 (11)	175 (10)	164 (10)	274 (10)	578 (11)	560 (12)
Missing, N (%)	1,168 (7)	90 (5)	113 (7)	160 (6)	434 (8)	371 (8)
Weight for gestational age, N (%)						
*Small*	1,445 (9)	185 (11)	154 (9)	228 (9)	493 (9)	385 (8)
*Appropriate*	11,845 (72)	1,255 (72)	1,218 (72)	1,927 (74)	3,915 (72)	3,530 (73)
*Large*	1,771 (11)	204 (12)	195 (12)	285 (11)	561 (10)	526 (11)
*Missing*	1,284 (8)	101 (6)	128 (8)	170 (7)	466 (9)	419 (9)

*AKI* Acute kidney injury, *IQR* Interquartile range, *pCr* Plasma creatinine, *CAKUT* Congenital anomalies of the kidney and urinary tract. *Community-acquired AKI: Outpatient clinic or the first day of admission. Hospital-acquired AKI: > 1 day of admission

### Additional analyses

**Fig. 3:**
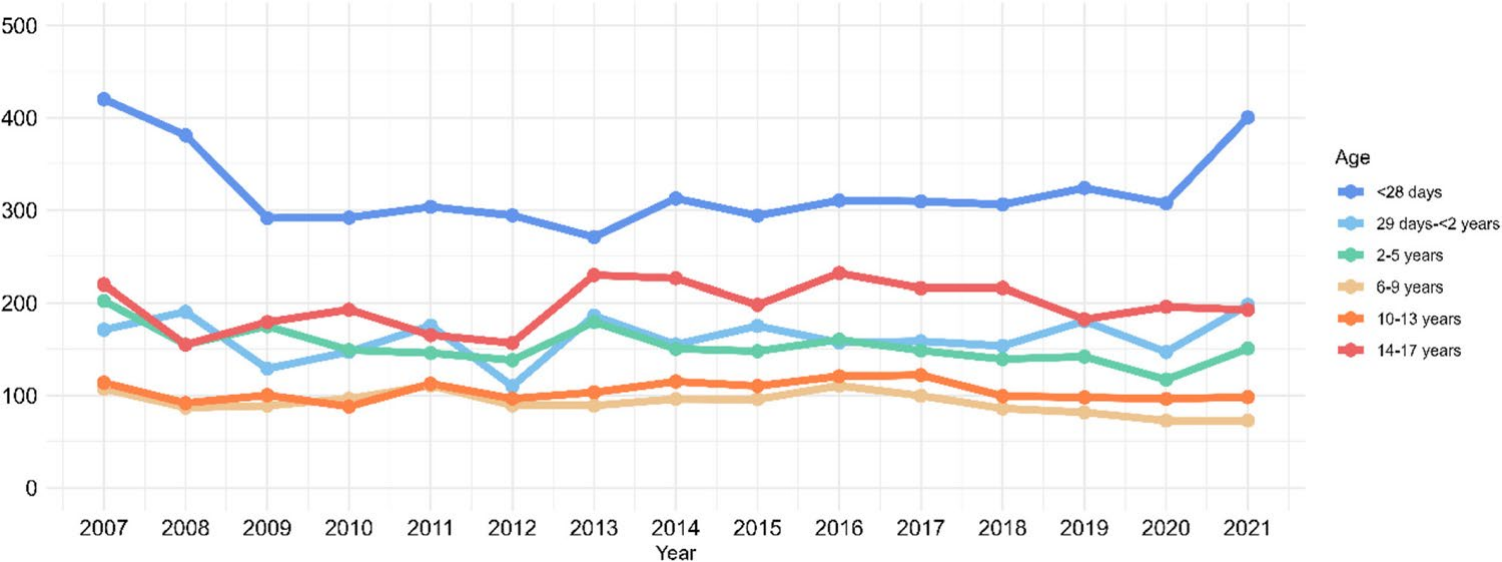
Annual AKI rate per 100,000 children according to age

**Fig. 4:**
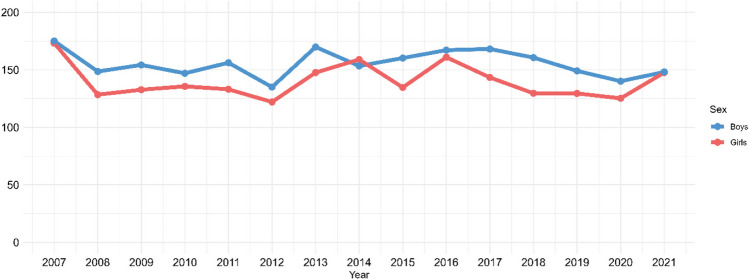
Annual AKI rate per 100,000 children according to sex

The number of children with at least one creatinine test per year for every 100,000 children covered by the laboratory databases, increased slightly over the 15-year study period (Supplementary Figure S1). In the age- and sex-stratified analyses, the average annual AKI rates were highest in children at the lower and upper age ranges, and among boys (Figs. 3 and 4). When including only the AKI episodes with a measured baseline pCr (*n* = 8,124 AKI episodes), the average annual AKI rate decreased to 74 (95% CI: 70–78) per 100,000 children (Supplementary Figure S2). When we restricted data to the study area covered by the laboratory databases throughout the study period (*n* = 7,865 AKI episodes), the average annual AKI rate was 144 (95% CI: 136–153) per 100,000 children (Supplementary Figure S3). After including additional AKI episodes defined by POCT (*n* = 1,001 AKI episodes), the average annual AKI rate increased to 160 (95% CI: 150–169) per 100,000 children (Supplementary Figure S4). In all sensitivity analyses, the annual rate remained stable throughout the study period.

## Discussion

In this population-based study, we found a stable rate of AKI among Danish children from 2007 to 2021. The combined rate for all types of AKI episodes showed only minor fluctuations across calendar years. Similarly, we found only minor changes in patient characteristics for pediatric AKI during the 15-year study period.

Our finding of no temporal change in AKI rate is consistent with some, but not all, studies in the adult setting [7–10, 32]. Studies on temporal changes are prone to changes in the underlying population or in surveillance including diagnostic coding, which may contribute to the increase in AKI rate reported in previous studies. Few other studies have reported the rate of pediatric AKI in population-based settings, but we are the first to describe the temporal trends in pediatric AKI rates. Two overlapping studies from Wales used electronic alerts to identify AKI episodes in children [5, 33]. Our overall finding on the rate of AKI confirmed the finding of the first study reporting an AKI incidence of 137 per 100,000 person-years among children < 18 years of age [5]. The second study excluded neonates and found a lower AKI incidence of 77.3 per 100,000 person-years [33]. In contrast to our study, both studies included all AKI episodes within a calendar year, but only the AKI episodes with a known baseline creatinine. Earlier studies based on hospital-recorded ICD codes of AKI, reported AKI incidences of 3.3 per 100,000 children and 3.9 per 1000 pediatric hospitalizations [3, 34]. A general observation in all previous studies, as well as in the current study, was the bimodal age distribution of AKI and the male predominance. The neonates and infants represent a unique population particularly susceptible to AKI. The immature state of their kidneys makes them sensitive to kidney damage from a variety of stressors. Until the age of two years, the kidneys are not considered fully developed [35]. Therefore, we restricted our analysis of children < 2 years of age to AKI episodes with known baseline pCr or AKI episodes confirmed by ICD-10 codes.

The strengths of our study include a population-based design and the utilization of the comprehensive Danish medical databases. Consequently, we have been able to incorporate longitudinal data not only on hospital-acquired AKI episodes but also on community-acquired episodes, which accounted for almost two-thirds of the episodes. Nevertheless, our study is not without limitations. First, as most previous studies, we did not have data to evaluate the urine output-based criteria of AKI as this is not routinely recorded. Instead, the reported AKI rates were solely based on the creatinine-based criteria of AKI, which could have led to an underestimation of the AKI rate. A multinational study found that the creatinine criteria failed to identify AKI in two thirds of children with low urine output [2]. While we may underestimate the absolute rate of AKI, the reported stable AKI rate should be robust as the proportion of children missed by our approach is unlikely to have changed during the study period. Second, the methods for estimating pCr changed over the study period [17]. In 2007, a few laboratories were still utilizing the Jaffe method, which is known for its non-specificity and propensity to overestimate pCr values. Consequently, when we applied the KDIGO criteria and the Pottel-age-based imputation model on pCr values derived from the Jaffe method, it may have overestimated the AKI rate in 2007. However, most laboratories switched to the enzymatic method before our study began. Third, when no baseline pCr was available, we required the AKI episodes to be confirmed by an ICD-10 code among children < 2 years and those with chronic kidney disease. This requirement was implemented due to the potential fluctuations in creatinine levels in these pediatric subgroups, making it challenging to establish a baseline creatinine. Therefore, this modification was deemed essential to improve specificity, albeit at the cost of reduced sensitivity. Fourth, we acknowledge that any change in pCr testing frequency may affect the annual AKI rate. Nevertheless, we found only a slight increase in the frequency of pCr testing throughout the 15-year study period, making it unlikely to have substantially influenced the observed annual AKI rate. Additionally, our findings demonstrated that the annual AKI rate is highly dependent on the AKI definition used. Specifically, when considering only AKI episodes with a known baseline pCr, the average annual AKI rate was halved. In contrast, including additional AKI episodes defined by POCT led to an approximate 10% increase in AKI rates. Fifth, we used routine clinical care data and acknowledge that the children with a pCr measurement may represent a selected population, who are likely to be less healthy than the broader pediatric population. As a result, it is likely that the rate of AKI at a population-level has been underestimated because clinicians are reluctant to draw a blood sample from a child without a clear indication. We sought to mitigate this by imputing a baseline pCr when it was unavailable. However, the use of the Pottel-age-based imputation model may also have resulted in some false-positive episodes of AKI being included in the analyses. Sixth, it is possible that changes in patient characteristics over time may not accurately reflect temporal changes in risk factors for pediatric AKI in the population. This limitation may arise due to the potential presence of collider stratification bias when analyzing baseline characteristics and risk factors conditional on AKI. To identify the actual risk factors for pediatric AKI, it would have been necessary to examine the prevalence of risk factors in the general population. However, the data required for such an analysis was not available. Furthermore, minor differences in patient characteristics such as ICD-10 coded comorbidities (e.g., kidney disease and birth defects) could be attributed to alterations in coding practice over time.

Our study provides valuable insight into the epidemiology of pediatric AKI in Denmark, but caution should be exercised in generalizing the rate to other countries with different healthcare systems and demographics. While our study did not identify any temporal changes in the pediatric AKI rate over the 15-year study period, it highlights the need for continued effort to prevent and manage AKI among children. Despite the introduction of KDIGO guidelines since 2012 and an increased awareness of AKI among clinicians, the rate of pediatric AKI has not decreased over time, suggesting that there is still room for improved prevention of AKI [1].

### Supplementary Information

Below is the link to the electronic supplementary material.

Graphical abstract (PPTX 358 KB)

Supplementary file2 (PDF 430 KB)
