# Supplementary material for: Fifteen-year temporal changes in rates of acute kidney injury among children in Denmark
Source: Pediatr Nephrol. 2023 Dec 18;39(6):1917–25. doi: 10.1007/s00467-023-06246-9 (PMC11026202; doi:10.1007/s00467-023-06246-9)
Supplement: Supplementary file 1 — Graphical abstract (PPTX 358 KB) [file 467_2023_6246_MOESM1_ESM.pptx]

## Slide 1
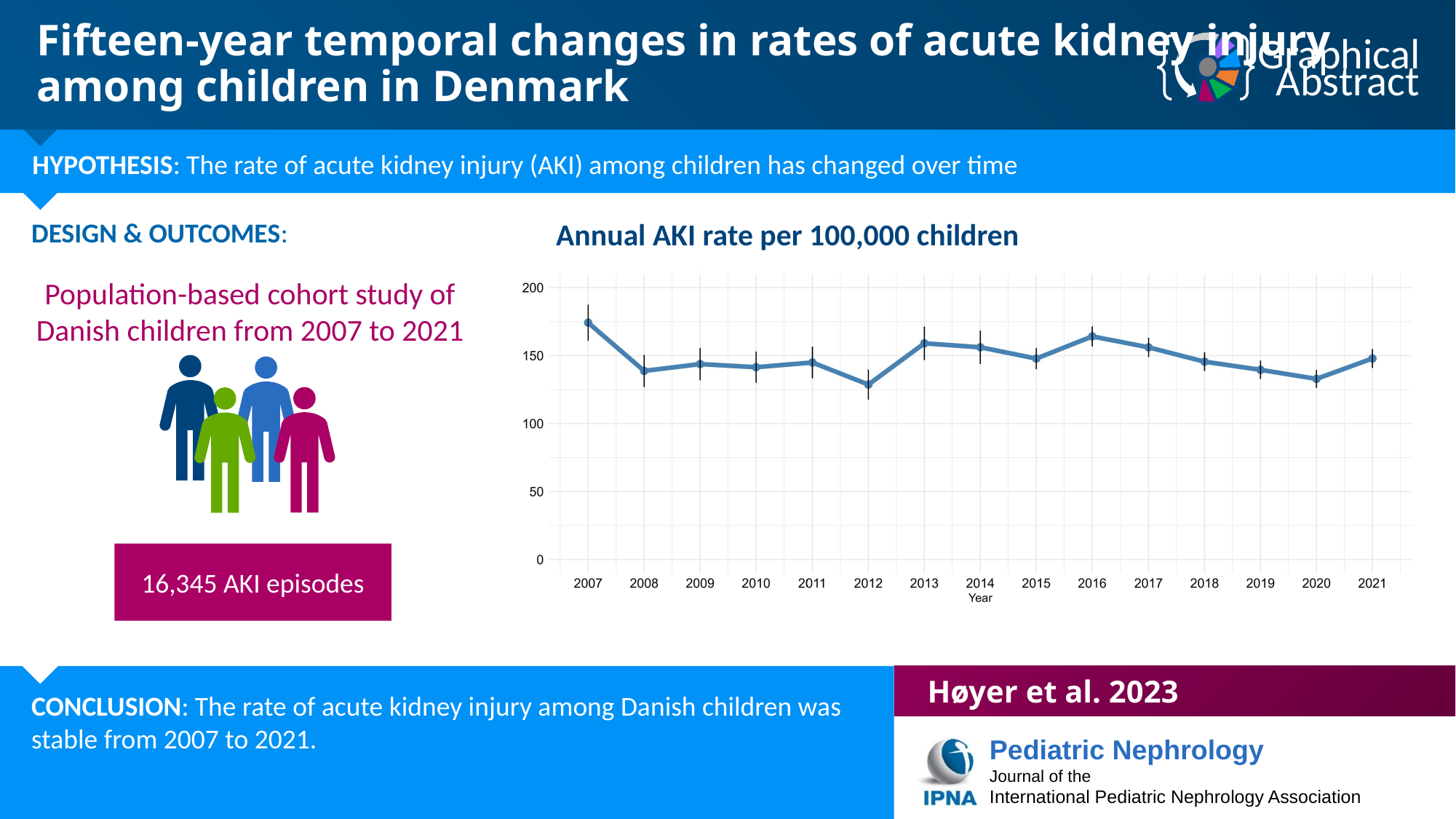

Fifteen-year temporal changes in rates of acute kidney injury
among children in Denmark
HYPOTHESIS: The rate of acute kidney injury (AKI) among children has changed over time
DESIGN & OUTCOMES:
Annual AKI rate per 100,000 children
Population-based cohort study of Danish children from 2007 to 2021
16,345 AKI episodes
Høyer et al. 2023
CONCLUSION: The rate of acute kidney injury among Danish children was stable from 2007 to 2021.
