# Supplementary material for: Fifteen-year temporal changes in rates of acute kidney injury among children in Denmark
Source: Pediatr Nephrol. 2023 Dec 18;39(6):1917–25. doi: 10.1007/s00467-023-06246-9 (PMC11026202; doi:10.1007/s00467-023-06246-9)
Supplement: Supplementary file 2 — Supplementary file2 (PDF 430 KB) [file 467_2023_6246_MOESM2_ESM.pdf]

Fifteen-year temporal changes in rates of acute kidney injury among children in Denmark

Sidse Høyer[1], Uffe Heide-Jørgensen[1], Simon Kok Jensen[1], Mette Nørgaard[1], Cara Slagle[2, 3, 4], Stuart Goldstein[2,3], Christian Fynbo Christiansen[1].

[1]Department of Clinical Epidemiology and Department of Clinical Medicine, Aarhus University Hospital and Aarhus University, Denmark.

[2]Center for Acute care Nephrology, Cincinnati Children's Hospital Medical Center, Ohio, US

[3]Department of Pediatrics, University of Cincinnati, Ohio, US

[4]Division of Neonatal and Pulmonary Biology, Cincinnati Children's Hospital Medical Center, Ohio, US

Corresponding author: Sidse Høyer
sh@clin.au.dk

Supplementary figures and tables

Supplementary figure S1: Number of children with at least one creatinine test per year

Supplementary figure S2: Annual AKI rate per 100,000 children, based on known baseline pCr only

Supplementary figure S3: Annual AKI rate per 100,000 children, restricted to study population with data from 2007

Supplementary figure S4: Annual AKI rate per 100,000 children, including POCT

Supplementary table S1: Pediatric and neonatal KDIGO guidelines

Supplementary table S2: ICD-codes for included comorbidity

Supplementary table S3: ICD10-codes for included recent risk factors

Supplementary table S4: ATC-codes for included potential nephrotoxic medications (only prescriptions filled at community pharmacies)

Supplementary figure S1: Number of children with at least one creatinine test per year

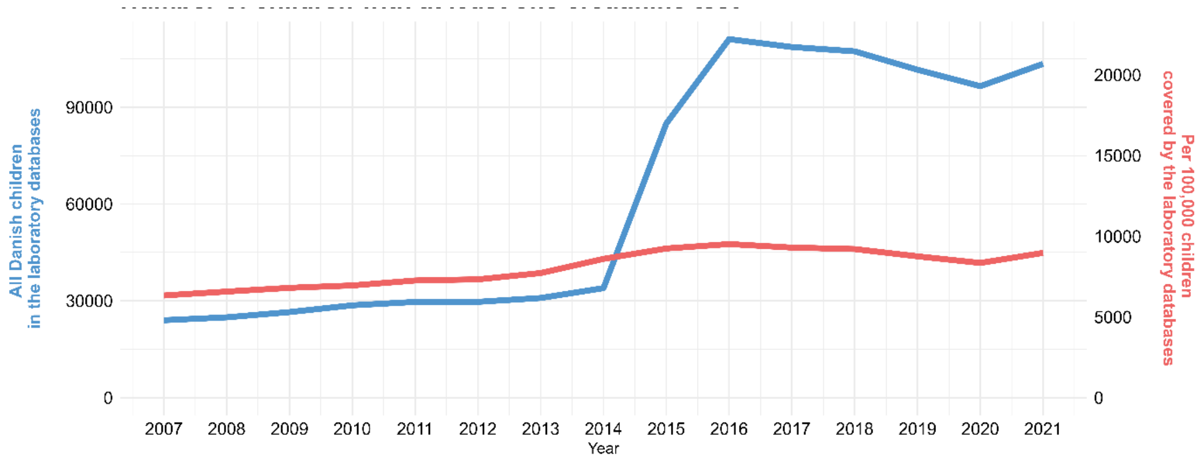

Supplementary figure S2: Annual AKI rate per 100,000 children, based on known baseline pCr only

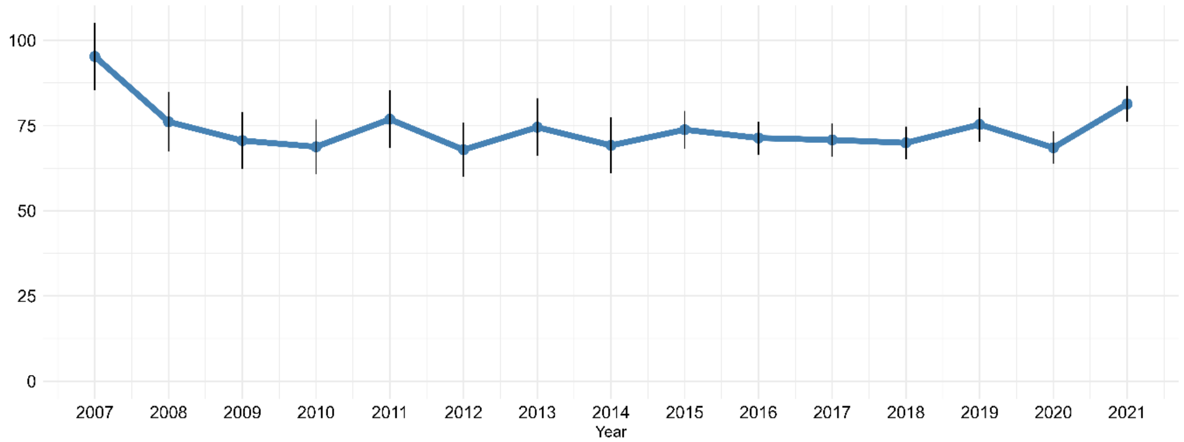

Supplementary figure S3: Annual AKI rate per 100,000 children, restricted to study population with data from 2007

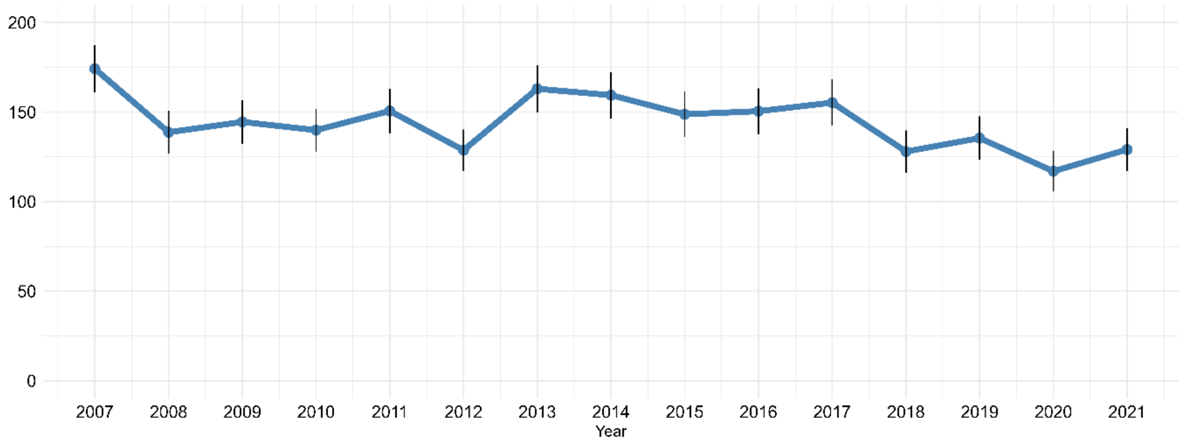

Supplementary figure S4: Annual AKI rate per 100,000 children, including POCT

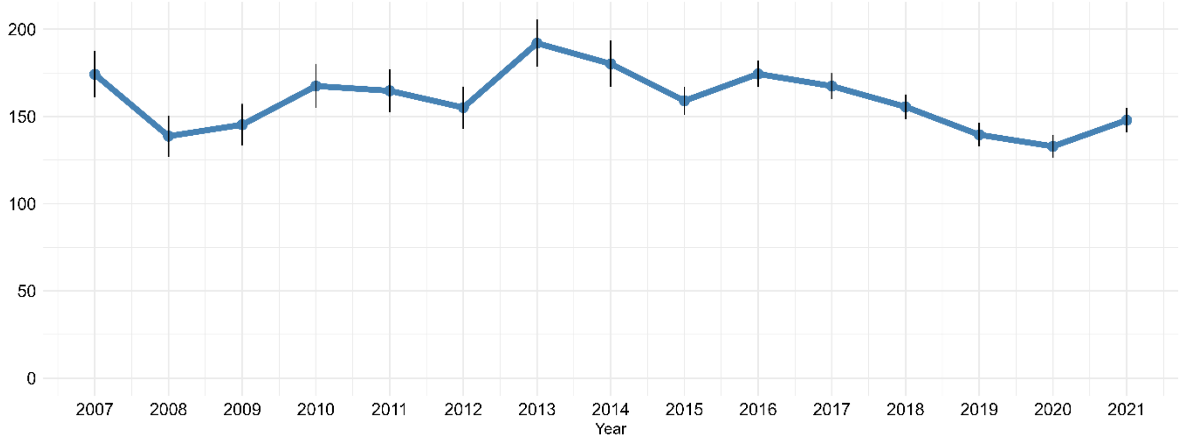

Supplementary table S1: Pediatric and neonatal KDIGO guidelines

Stage	Pediatric KDIGO criteria		Neonatal modified KDIGO criteria	
	Serum creatinine	Urine output	Serum creatinine	Urine output
**1**	1.5-1.9 times baseline within 7 days<br>OR<br>≥26.5 μmol/L increase within 48 h	<0.5 mL/kg/h for 6-12 h	1.5-1.9 times baseline* within 7 days<br>OR<br>≥26.5 μmol/L increase within 48 h	>0.5 and ≤1 mL/kg/h over 24 h
**2**	2.0-2.9 times baseline	<0.5 mL/kg/h for ≥12 h	2.0-2.9 times baseline*	>0.3 and ≤0.5 mL/kg/h over 24 h
**3**	≥3.0 times baseline<br>OR<br>Increase in serum creatinine to ≥353.6 μmol/L<br>OR<br>Initiation of renal replacement therapy<br>OR<br>Decrease in eGFR to <35 mL/min per 1.73m^2	<0.3 mL/kg/h for ≥24 h<br>OR<br>Anuria for ≥12 h	≥3.0 times baseline*<br>OR<br>Increase in serum creatinine to ≥221 μmol/L<br>OR<br>Initiation of renal replacement therapy	≤0.3 mL/kg/h over 24 h

* Baseline serum creatinine is the lowest previous value.

Supplementary table S2: ICD-codes for included comorbidities

**ICD10-codes for included comorbidities with 5 years lookback**	
Cancer	
C00-D99 (Except C43)	Malignant neoplasms (Except malignant melanoma of skin)
Cardiac disease	
I25	Chronic ischemic heart disease
I42	Cardiomyopathy
I43	Cardiomyopathy in diseases classified elsewhere
I44	Atrioventricular and left bundle-branch block
I45	Other conduction disorders
I46	Cardiac arrest
I47	Paroxymal tachycardia
I48	Atrial fibrillation and flutter
I49	Other cardiac arrhythmias
I50	Heart failure
Diabetes	
E10	Type 1 diabetes mellitus
E11	Type 2 diabetes mellitus
E12	Malnutrition-related diabetes mellitus
E13	Other specified diabetes mellitus
E14	Unspecified diabetes mellitus
Hypertension	
I10	Essential (primary) hypertension
I11	Hypertensive heart disease
I12	Hypertensive renal disease
I13	Hypertensive heart and renal disease
I15	Secondary hypertension
Kidney disease	
N03	Chronic nephritic syndrome
N05	Unspecified nephritic syndrome
N07	Hereditary nephropathy, not elsewhere classified
N08	Glomerular disorders in diseases classified elsewhere
N11	Chronic tubulo-interstitial nephritis
N150	Balkan nephropathy
N16	Renal tubule-interstitial disorders in diseases classified elsewhere
N18	Chronic kidney disease
N19	Unspecified kidney failure
E102	Type 1 diabetes mellitus with renal complications
E112	Type 2 diabetes mellitus with renal complications
E122	Malnutrition-related diabetes mellitus with renal complications
E132	Other specified diabetes mellitus with renal complications
E142	Unspecified diabetes mellitus with renal complications
I12	Hypertensive renal disease
I13	Hypertensive heart and renal disease
Liver and bowel disease	
B18	Chronic viral hepatitis
K50	Crohn disease
K51	Ulcerative colitis
K528	Other specified noninfective gastroenteritis and colitis
K713	Toxic liver disease with chronic persistent hepatitis
K714	Toxic liver disease with chronic lobular hepatitis

K715	Toxic liver disease with chronic active hepatitis
K721	Chronic hepatic failure
K729	Hepatic failure, unspecified
K73	Chronic hepatitis, not elsewhere classified
K74	Fibrosis and cirrhosis of liver
Pulmonary disease	
J40	Bronchitis, not specified as acute or chronic
J41	Chronic and mucopurulent chronic bronchitis
J42	Unspecified chronic bronchitis
J43	Emphysema
J44	Other chronic obstructive pulmonary disease
J45	Asthma
J47	Bronchiectasis
J953	Chronic pulmonary insufficiency following surgery
J961	Chronic respiratory failure
J982	Interstitial emphysema
**ICD8 and ICD10-codes for included comorbidities with lifelong lookback**	
Birth defects	
740-759<br>Q00-Q99	Congenital anomalies<br>Congenital malformations, deformations and chromosomal abnormalities
Congenital anomalies of the kidney and urinary tract (CAKUT)	
753<br>Q60-Q64	Congenital anomalies of urinary system<br>Congenital malformations of the urinary tract
Congenital heart disease	
746-747	Congenital anomalies of the heart and circulatory system
Q20	Congenital malformations of cardiac chambers and connections
Q21	Congenital malformations of cardiac septa
Q22	Congenital malformations of pulmonary and tricuspid valves
Q23	Congenital malformations of aortic and mitral valves
Q24	Other congenital malformations of heart
Q25	Congenital malformations of great arteries

Supplementary tableS3: ICD10-codes for included recent risk factors

**ICD10-codes for included recent risk factors with 3 months lookback**	
Any diagnosed kidney disease (acute or chronic)	
N00-N08	Glomerular diseases
N10-N16	Renal tubulo-interstitial diseases
N17-N19	Renal failure
N20	Calculus of kidney and ureter
N25-N29	Other disorder of kidney and ureter
Any infection	
A00-A09	Intestinal infectious disease
A15-A19	Tuberculosis
A20-A28	Certain zoonotic bacterial diseases
A30-A49	Other bacterial diseases
A50-A64	Infections with a predominantly sexual mode of transmission
A70-A74	Other diseases caused by chlamydia
A75-A79	Rickettsioses
A80-A89	Viral infections of the central nervous system
A92-A99	Arthropod-borne viral fevers and viral hemorrhagic fevers
B00-B09 (except B07-B09)	Viral infections characterized by skin and mucous membrane lesions (except viral warts and unspecified viral infection characterized by skin and mucous membrane lesions)
B15-B19	Viral hepatitis
B20-B24	Human immunodeficiency virus [HIV] disease
B25-B34	Other viral diseases
B35-B49 (except B35-B36)	Mycoses (except dematophytosis and other superficial mycosis)
B50-B64	Protozoal diseases
B99	Other infectious diseases
G00-G09 (except G03 and G09)	Infections in the central nervous system (except meningitis due to other and unspecified causes and sequelae of inflammatory diseases of the central nervous system)
I301	Infective pericarditis
I33	Acute and subacute endocarditis
I40	Acute myocarditis
J00-J06 (except J00)	Acute upper respiratory infections (except nasopharyngitis [common cold])
J09-J18	Influenza and pneumonia
J20-J22	Other acute lower respiratory infections
J36	Peritonsillar abscess
J390	Retropharyngeal and parapharyngeal abscess
J391	Other abscess of pharynx
J440	Chronic obstructive pulmonary disease with acute lower respiratory infection
J85-J86	Suppurative and necrotic conditions of lower respiratory tract
K35	Acute appendicitis
K570	Diverticular disease in the small intestine with perforation and abscess
K572	Diverticular disease in the large intestine with perforation and abscess
K574	Diverticular disease in both small and large intestine with perforation and abscess
K578	Diverticular disease of intestine, part unspecified with perforation and abscess
K61	Abscess of anal and rectal regions
K630	Abscess of intestine
K631	Perforation of intestine (nontraumatic)

K65	Peritonitis
K750	Abscess of liver
K751	Phlebitis of portal vein
K810	Acute cholecystitis
K830	Cholangitis
M00	Pyogenic arthritis
M01	Direct infectious of joint in infectious or parasitic diseases classified elsewhere
M462	Osteomyolitis of vertebra
M463	Infection of intervertebral disc (pyogenic)
M465	Other infective spondylopathies
M60	Myocitis
M726	Necrotizing fasciitis
M86	Osteomyelitis
N10	Acute tubule-interstinal nephritis
N300	Acute cystitis
N309	Cystitis, unspecified
N390	Urinary tract infection, site not specified
N410	Acute prostatitis
N412	Abscess of prostate
N413	Prostatocystitis
N418	Other prostatitis
N45	Orchitis and epididymitis
N70-77	Inflammatory disease of the female pelvic organs
O030	Spontaneous abortion: incomplete, complicated by genital tract and pelvic infection
O035	Spontaneous abortion: complete or unspecified, complicated by genital tract and pelvic infection
O080	Genital tract and pelvic infection following abortion and actopic and molar pregnancy
O23	Infection of genitourinary tract in pregnancy
O85	Puerperal sepsis
O86	Other puerperal infections
P23	Congenital pneumonia
P35-P39	Infections specific for the perinatal period
R572	Septic shock
T826	Infections and inflammatory reaction due to cardiac valve prosthesis
T827	Infections and inflammatory reaction due to other cardiac and vascular devices, implants and grafts
T835	Infections and inflammatory reaction due to prosthetic devices, implants and grafts in urinary tract
T836	Infections and inflammatory reaction due to prosthetic devices, implants and grafts in genital tract
T845	Infections and inflammatory reaction due to internal joint prosthesis
T846	Infections and inflammatory reaction due to internal fixation device [any site]
T847	Infections and inflammatory reaction due to other internal orthopaedic prosthetic devices, implants and grafts
T857	Infections and inflammatory reaction due to other internal prosthetic devices, implants and grafts
Sepsis	
A021	Salmonella sepsis
A207	Septicaemic plague
A227	Anthrax sepsis
A267	Erysipelothrix sepsis

A282B	Yersinia sepsis
A327	Listerial sepsis
A392A	Meningococcal sepsis
A40	Streptococcal sepsis
A41	Other sepsis
A427	Actinomycotic sepsis
A548G	Gonococcal sepsis
B377	Candidal sepsis
J950A	Sepsis from tracheostomy stoma
O080U	Sepsis after abortion
O753A	Sepsis during labour
O85	Puerperal sepsis
P36	Bacterial sepsis of newborn
T802D1	CVC-related sepsis
T814D	Postoperative sepsis
T880A	Sepsis after vaccination
Surgery	
K<br>(Except KT and KU)	Surgery (except minor surgical procedures and endoscopies)

Supplementary table S4: ATC-codes for included potential nephrotoxic medications (only prescriptions filled at community pharmacies)

**Drug**	**ATC**
Analgesia	
Acetaminophen	N02BE, N02AJ01, N02AJ06, N02AJ13, N02AJ17
Aspirin	B01AC06, B01AC56, C07FX02, C07FX03, C07FX04, C10BX01, C10BX02, C10BX04, C10BX05, C10BX06, C10BX08, C10BX12, M01BA03, N02AJ02, N02AJ07, N02AJ18, N02BA01, N02BA51, N02BA71
Nonsteroidal anti-inflammatory drugs	M01A
Antidepressants/mood stabilizers	
Amitryptyline, fluoxetine, doxepin	N06AA09, N06CA01, N06AB03, N06CA03, N06AA12
Lithium	N05AN
Antidiabetics	
Chlorpropamide	A10BB02
Tolbutamide	A10BB03, V04CA01
Antiepileptics	
Topiramate	N03AX11
Zonisamide	N03AX15
Antihistamines	
Diphenhydramine, doxylamine	R06AA02, R06AA09, R06AA59
Antimicrobials	
Acyclovir, valaciclovir	J05AB01, J05AB11
Aminoglycosides	J01G
Amphotericin B	A07AA07, J02AA01
Beta lactams (penicillins, cephalosporins)	J01CA, J01CE, J01CF, J01CR
Colistine	A07AA10, J01XB01
Foscarnet	J05AD01
Ganciclovir, valganciclovir	J05AB06, J05AB14
Pentamidine	P01CX01
Quinolones	J01M
Rifampin	J04AB02, J04AM
Sirolimus	L01EG01, L04AA10
Sulfazalazine	A07EC01
Sulfonamides	J01EB, J01EC, J01ED, J01EE
Tetracycline	J01AA, J01RA08,
Vancomycin	A07AA09, J01XA01
Antiretrovirals	
Adefovir, cidofovir, tenofovir	J05AF08, J05AB12, J05AB17, J05AF07, J05AF13, J05AR03, J05AR06, J05AR07, J05AR08, J05AR09, J05AR11, J05AR12, J05AR17, J05AR18, J05AR19, J05AR20, J05AR22, J05AR24, J05AR27
Indinavir	J05AE02
Antiuremics	
Allopurinol	M04AA01, M04AA51
Probenecid	M04AB01
Benzodiazepines	
Benzodiazepines	N03AE, N05BA, N05CD, N05CF

Calcineurin inhibitors	
Cyclosporine	L04AD01
Tacrolimus	L04AD02
Cardiovascular agents	
Angiotensin-converting enzyme inhibitors	C09AA, C09B
Angiotensin receptor blockers	C09C, C09D, C09DB, C09DX
Clopidogrel, ticlopidine	B01AC04
Statins	C10AA, C10BA, C10BX
Chemotherapeutics	
Carmustine, semustine	L01AD01, L01AD03
Cisplatin, carboplatin	L01XA01, L01XA02
Cyclophosphamide, ifosfamid	L01AA01, L01AA06
Interferon-alfa	L03AB01, L03AB04, L03AB05, L03AB06
Methotrexate	L01BA01, L04AX03
Mitomycin-C	L01DC03
Contrast agents	
Contrast agents	V08
Diuretics	
Diuretics in combination with other drugs	C02L, C03E, C07CA, C07D, C08G, C09BA, C09DA
Loops	C03C
Thiazides	C03A
Triamterene	C03DB02
Drugs of abuse	
Cocaine, heroin, ketamine, methadone, methampetamine	N01BC01, N01AX03, N07BC02, N02AC52, N06BA03
H2 receptor blocker	
Cimetidine, ranitidine	A02BA01, A02BA51, A02BA02, A02BA07
Proton pump inhibitors	
Lansoprazole, omeprazole, pantoprazole	A02BC03, A02BC53, A02BD, A02BC01, A02BC05, A02BD01, A02BD05, A02BD06, A02BD16, M01AE52, A02BC02, A02BD04, A02BD11
Others	
Acetazolamide	S01EC01
Gold therapy	M01CB, V10AX06
Dapsone	J04BA02
Haloperidol	N05AD01
Mesalazin	A07EC02
Pamidronate	M05BA03
Penicillamine	M01CC01
Phenytoin	N03AB
Quinine	P01BC01
Zoledronate	M05BA08, M05BB08
